# m6A RNA methylation dynamics during in vitro maturation of cumulus-oocyte complexes derived from adult or prepubertal sheep

**DOI:** 10.1007/s10815-025-03444-2

**Published:** 2025-03-18

**Authors:** Chiara Cosseddu, Sara Succu, Adele Frau, Francesca Mossa, Sylvia Virginie Versace, Tiziana A. L. Brevini, Sergio Ledda, Daniela Bebbere

**Affiliations:** 1 Department of Veterinary Medicine, Obstetrics and Gynecology Clinics, University of Sassari, 07100 Sassari, Italy; 2 Department of Veterinary Medicine, Anatomy, University of Sassari, 07100 Sassari, Italy; 3 Department of Veterinary Medicine, Veterinary Teaching Hospital, University of Sassari, 07100 Sassari, Italy; 4 Laboratory of Biomedical Embryology, Department of Veterinary Medicine and Animal Science and Center for Stem Cell Research, University of Milano, 26900 Lodi, Italy

**Keywords:** Oocyte, Cumulus cells, Post-transcriptional regulation, m6A methylation-related proteins, Developmental competence, Epitranscriptomics

## Abstract

**Purpose:**

N6-methyladenosine (m6A) is the most prevalent base epigenetic modification within eukaryotic mRNAs. It participates in post-transcriptional regulation, including maternal RNA maintenance and decay in mouse oocytes and during maternal-to-zygotic transition. The landscape in other mammalian species remains largely unexplored. The present work analyzed m6A dynamics in sheep cumulus oocyte complexes (COCs), during in vitro maturation. To explore potential relationships with oocyte developmental competence, a previously established model consisting of oocytes derived from adult and prepubertal sheep was adopted.

**Methods:**

m6a dynamics were analyzed in terms of m6A RNA methylation abundance in cumulus cells (CCs) by colorimetric assay and expression of key m6A methylation-related proteins (*METTL3, METTL14, METTL16, VIRMA, YTHDC1, YTHDC2, YTHDF2, YTHDF3, ALKBH5*, and *FTO*) in both cumulus cells and oocytes by real-time PCR.

**Results:**

We report the dynamics of m6A in sheep COCs, and reveal alterations in both oocytes and cumulus cells derived from prepubertal donors. These changes were observed in terms of m6A RNA methylation levels and transcript dynamics of several m6A methylation-related proteins. Notably, our study shows that dysregulations occur after IVM.

**Conclusion:**

Overall, this work describes for the first time the dynamics of m6A in sheep COCs and uncovers the involvement of m6A RNA methylation in oocyte developmental potential.

## Introduction

In metazoans, oocyte maturation, fertilization and early embryo development occur in the absence of de novo transcription; as a consequence, these events rely on the mRNAs stored during oocyte growth for protein synthesis. Transcription resumes upon activation of the embryo genome (EGA), when maternal mRNAs are degraded and replaced by the molecules synthesized by the embryo [1, 2].

During the transcriptional silence, gene regulation depends completely on post-transcriptional mechanisms, which cooperate to achieve a precise and timely recruitment of maternal RNAs for translation or degradation [3]. This complex and multi-layered process is key to successful oocyte maturation, fertilization, and embryonic development, although it remains incompletely characterized. It is known that stabilization and recruitment of maternal mRNAs involve regulation of the length of the poly(A) tails [4], the presence of specific sequences in transcript 3′UTR and the action of various RNA-binding proteins and microRNAs [5]. An additional but much less characterized level of post-transcriptional regulation is mRNA editing.

Similarly to DNA, RNA is also dynamically modified by the attachment of chemical groups [6]. N6-methyladenosine (m6A) is a conserved and abundant base epigenetic modification of mRNA, occurring mainly on messenger and long non-coding RNAs [7]. m6A marks are non-randomly located on mRNAs, being found predominantly in the 3′ untranslated regions and near stop codons [8]. Modulation of m6A levels is involved in the regulation of mRNA stability, translation, and splicing in both normal and pathological conditions [8]. In the oocyte and early embryo, m6A acts a protective factor to ensure the stability of maternal RNAs and determine their fate, thereby affecting oocyte developmental competence [9, 10].

**Fig. 1:**
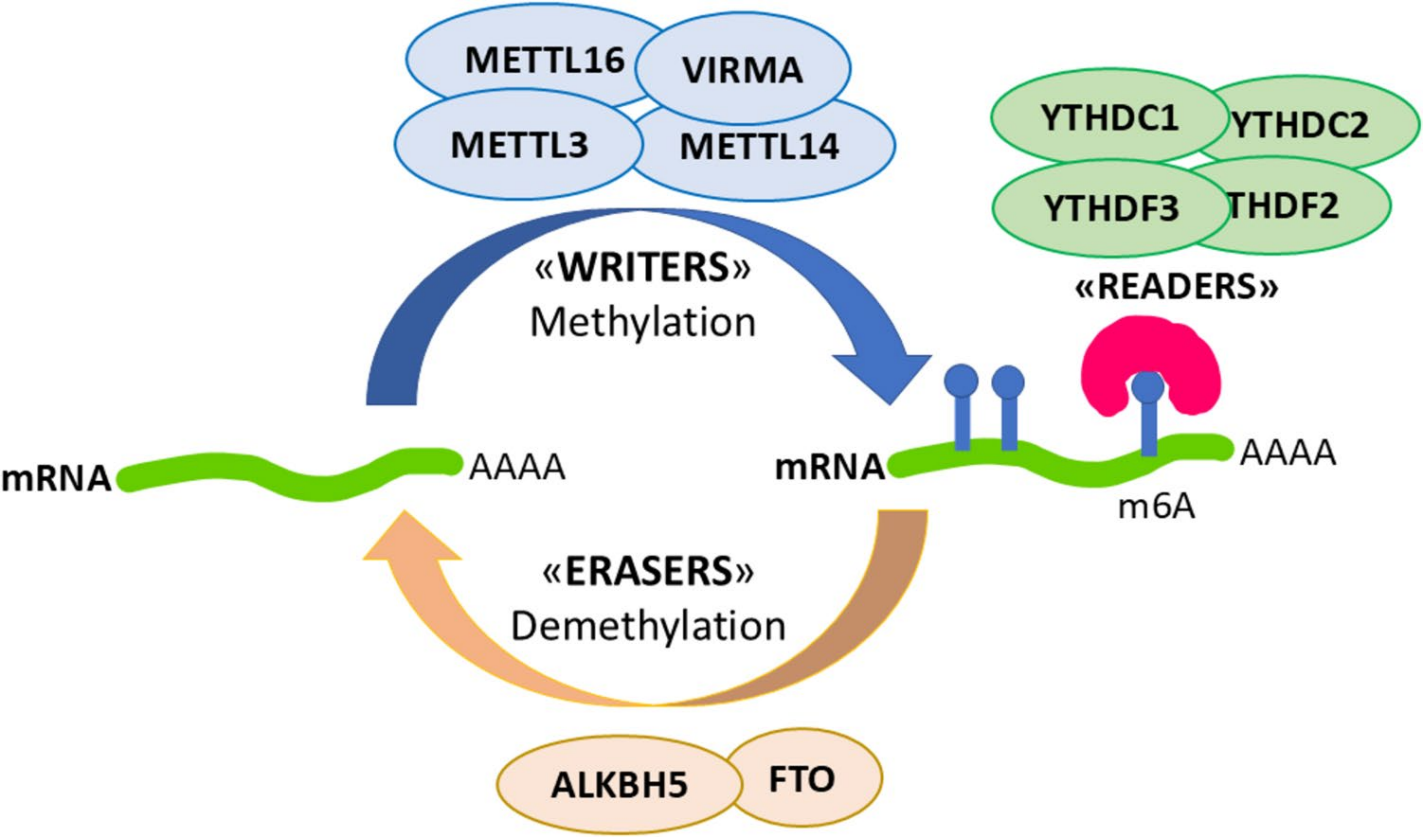
Representative scheme of the m6A methylation-related proteins analyzed in the present study: “writers” (*METTL3 METTL14*,* METTL16*, and *VIRMA*), “readers” (*YTHDC1*, *YTHDC2*, *YTHDF2*, and *YTHDF3*), and “erasers” (*ALKBH5* and *FTO*)

RNA methylation dynamics are controlled by three different groups of proteins, which are defined as writers (enzymes that add the methyl group to the N6 position of adenosine, e.g., METTL3 and METTL4), erasers (enzymes that remove the methyl group, e.g., FTO and ALKBH5), and readers (proteins that bind to m6A marks and activate downstream events, e.g., YTHDF and YTHDC families; Fig. 1). Modulation of the activity of these proteins regulates RNA methylation, thereby affecting RNA splicing, export, decay, stability, and translation [8].

Research into RNA methylation dynamics during oogenesis and early embryo development is limited; yet, its pivotal role in female fertility has recently emerged [11]. m6A RNA methylation was described in mouse oocytes and pre-implantation embryos, and was seen to be altered in parthenogenotes [12]. Roles of m6A in folliculogenesis and oocyte maturation were explored in mouse, human, and swine [13–17], while functional studies in mice showed the indispensable functions of m6A modulators in oogenesis and embryo development [9] ( Table 1). More importantly, roles in regulation of transcript dosage were demonstrated for the m6A reader YTHDF2 [3] and for the eraser ALKBH5 [10] during mouse oocyte maturation. m6A dynamics during oocyte maturation and embryogenesis in ruminants remain largely unexplored [18, 19].

**Table 1:** Effects of null mutations of the analyzed m6A “writers,” “readers,” and “erasers” on female fertility (***data not available)

Type	Gene	Proposed function	Effects of null mutations on female fertility
**m6A writers**	*Mettl3*	mRNA stability and decay [20, 21]	Defects in polar body extrusion and increased aneuploidy rate [21]
	*Mettl14*	mRNA stability of histone modifiers [22]	Embryonic or peri-natal death [22]
	*Mettl16*	mRNA splicing and destabilization [23]	Failure of embryo implantation [24]
	*Virma*	Alternative splicing [25]	Abnormal chromatin organization and defects in oocyte maturation [16]
**m6A readers**	*Ythdc1*	mRNA splicing [26]; mRNA nuclear export [27]	Embryo arrest at early-post implantation stages [14]
	*Ythdc2*	***	Arrested oogenesis [28]
	*Ythdf2*	mRNA deadenylation [7]	Two cell-stage embryo arrest and cytokinesis defects [3]
	*YTHDF3*	mRNA translation [29]	***
**m6A erasers**	*Alkbh5*	RNA metabolism and mRNA export [30]	Compromised oocyte maturation; embryo developmental arrest [10]
	*FTO*	pre-mRNA processing and alternative splicing [31]	***

In the present work, we characterized m6A in cumulus-oocyte complexes (COCs) during in vitro maturation in sheep. To explore potential relationships with oocyte developmental competence, we adopted a previously established model consisting of oocytes derived from adult and prepubertal sheep, which show high and low developmental competence, respectively (reviewed in [32]). Oocytes derived from adult animals are fully grown and contain all the necessary molecules for maturation, fertilization, and the early stages of pre-implantation development. In contrast, oocytes from prepubertal donors have not completed their growth; however, they can still be matured, fertilized, and develop into an embryo in vitro, resulting in live offspring, albeit at lower rates compared to adult gametes [32, 33]. This ability suggests that at least some oocytes derived from prepubertal donors contain a subset of molecules sufficient to support complete development, making them valuable for analyzing the molecular pathways involved in oocyte developmental plasticity.

Morphological, cellular, biochemical, and molecular studies have confirmed the differential competence of gametes derived from adult and prepubertal donors in several aspects [32, 34–37]. Interestingly, we showed that in vitro-produced embryos derived from prepubertal donors exhibit a delay in maternal transcript degradation prior to embryo genome activation (2–4 cell stage [38]). We propose that the observed delay in maternal transcript degradation in low competence early embryos is due to altered post-transcriptional regulating mechanisms in the oocyte, including m6A modulation.

On this basis, in the present work, we tested the hypothesis that low competence oocytes present alterations in m6A methylation dynamics. To this aim, we analyzed the m6A methylation of sheep COCs with different developmental competence before and after in vitro maturation in terms of m6A RNA methylation abundance in cumulus cells and expression of ten m6A methylation-related proteins in both cumulus cells and oocytes.

## Materials and methods

All chemicals were purchased from Sigma Chemical CO. (St. Louis, MO) unless otherwise stated.

### Oocyte recovery and in vitro maturation

Oocytes were recovered from the ovaries of adult (4–5 years of age, body weight 35–40 kg) and prepubertal (4 weeks of age, body weight 6–10 kg) sheep, collected at a local slaughterhouse and transported to the laboratory within 1 h in Dulbecco’s phosphate-buffered saline (PBS) with antibiotics. After washing in fresh medium, ovaries were sliced using a micro-blade, and the follicle content was released in TCM-199 medium (with Earle’s salts bicarbonate) supplemented with 25 mmol HEPES, 0.1 g/L penicillin, 0.1 g/L streptomycin, and 0.1% (w/v) polyvinyl alcohol. Cumulus oocyte complexes (COCs) derived from both prepubertal and adult donors, showing several intact cumulus cell layers and a compact cytoplasm, were selected and matured in vitro (IVM) in TCM 199 supplemented with 10% heat-treated oestrus sheep serum (OSS), 1 IU/mL FSH and 1 IU/mL LH (Pergonal, Serono Italy), 8 mg/mL of pyruvate, and 100 μM cysteamine. Then, 30–35 COCs were cultured for 24 h in 5% CO_2_ in air at 38.5 °Cin four-well Petri dishes (Nunclon; Nalge Nunc, Roskilde, 100 Denmark) with 600 μL maturation medium, layered with 300 μL mineral oil. After IVM, oocytes underwent cumulus cells removal by gentle pipetting, and only oocytes presenting compact cytoplasm and the first polar body were selected. Oocytes and cumuluscells (CCs) at germinal vesicle stage (GV) or at metaphase II (MII) stage were separately stored in RNAprotect® Cell Reagent (Qiagen) and kept at − 20 °C until further analysis.

### Gene expression analysis

#### RNA isolation and reverse transcription

Total RNA was isolated from pools of ten denuded oocytes at GV or MII stage and from cumulus cells derived from pools of 20 COCs of adult or prepubertal animals. Five biological replicates were analyzed for each four experimental groups for oocyte (*n* = 5 pools of 10 GV oocytes derived from adults donors; *n* = 5 pools of 10 GV oocytes derived from prepubertal donors; *n* = 5 pools of 10 MII oocytes derived from adults donors; *n* = 5 pools of 10 MII oocytes derived from prepubertal donors) and cumulus cells (*n* = 5 pools of 20 GV COCs derived from adults donors; *n* = 5 pools of 20 GV COCs derived from prepubertal donors; *n* = 5 pools of 20 MII COCs derived from adults donors; *n* = 5 pools of 20 MII COCs derived from prepubertal donors).

Total RNA was isolated from the pools of oocytes or cumulus cells with the kit RNeasy® Micro (Qiagen, Hilden, Germany) following manufacturer’s instructions.

Five pg of luciferase mRNA (Promega) were added to each oocyte biological replicate prior to RNA extraction to account for RNA loss during the isolation process. During the procedure, RNA was treated with DNase I to exclude any potential genomic DNA contamination. Isolated RNA was eluted in 15 μl RNase-free water.

The RNA isolated from oocytes was immediately used for reverse transcription–polymerase chain reaction (RT-PCR). The RNA isolated from cumulus cells was spectroscopically checked with NanoDrop Lite (Fisher Scientific S.A.S., Illkirch Cedex, France) in terms of quantity and purity. A total of 100 ng RNA was used for cDNA synthesis per CC replicate.

Reverse-transcription was performed in a final volume of 20 μl, consisting of 50 mM Tris–HCl (pH 8.3), 75 mM KCl, 3 mM MgCl2, 5 mM DTT, 1 mM dNTPs, 2.5 μM random hexamer primers, 20 U RNase OUT, and 100 U SuperScript III RT (all purchased at Invitrogen Corporation, Carlsbad, CA). The reaction tubes were incubated at 25 °C for 10 min, then at 42 °C for 1 h, and finally at 70 °C for 15 min to inactivate the reaction. One tube without RNA and one with RNA, but no reverse transcriptase, were analyzed as negative controls.

To quantify the RNA recovery rate for oocyte samples, 5 pg of luciferase mRNA (not subjected to RNA isolation) were subjected to cDNA synthesis as well and subsequently quantified by real-time PCR.

#### Real-time polymerase chain reaction

Gene expression analysis by real-time PCR was performed and is described according to MIQE guidelines [39] and in line with recent recommendations [40].

All primers were designed using Primer3 version 0.4.0 (http://frodo.wi.mit.edu/primer3/) to be intron-spanning as a further control to prevent amplification of potential trace genomic DNA, in the unlikely event of incomplete DNA digestion by DNase I (Table 2).

**Table 2:** Primers used for gene expression analysis by real-time PCR. Bps , base pairs

Gene	Symbol	Sequence	Accession number	Annealing temperature	Size (bps)
Actin β	*ACTB*	F: 5′ ttcctgggtatggatcctg 3′ R: 5′ ggtgatctccttctgcatcc 3′	NM_001009784	60 °C	162
AlkB homolog 5, RNA Demethylase	*ALKBH5*	F: 5′cgtgtccttcttcagcgact 3′ R: 5′ctgaggatgatgaccgctct 3′	XM_004023544.5	59 °C	198
FTO alpha-ketoglutarate dependent dioxygenase	*FTO*	F: 5′catggcttccctacctgacc 3′ R: 5′ctggcagcttctcggaga 3′	XM_027977360.2	60 °C	91
Luciferase reporter vector pXP2 *SA *PS	*LUC*	F: 5′gctgggcgttaatcagagag 3′ R: 5′gtgttcgtcttcgtcccagt 3′	AF093685	58 °C	151
N6-adenosine-methyltransferase complex catalytic subunit 3	*METTL3*	F: 5′gcccactgatgctgtgtcta 3′ R: 5′gagcggcaaacttctgtagg 3′	XM_004010362.5	60 °C	98
N6-adenosine-methyltransferase non catalytic subunit 14	*METTL14*	F: 5′ctaaactgcgggagctcatc 3′ R: 5′attagcagtgatgccggtct 3′	XM_004009592.5	60 °C	182
Methyltransferase 16, RNA N6-adenosine	*METTL16*	F: 5′actctgacaaaagtaccctccg 3′ R: 5′tgctccacattcttctttgcg 3′	XM_004012555.4	60 °C	154
Ribosomal protein L19	*RPL19*	F: 5′caactcccgccagcagat 3′ R: 5′ccgggaatggacagtcaca 3′	XM_004012836	56 °C	127
Succinate dehydrogenase complex flavoprotein, subunit A	*SDHA*	F: 5′ catccactacatgacggagca 3′ R: 5′ atcttgccatcttcagttctgcta 3′	XM_012125144	60 °C	90
Vir like m6A methyltransferase associated	*VIRMA*	F: 5′atcagctgttggccatgttt 3′ R: 5′acgtcgctggaagactgaac 3′	XM_004011833.5	60 °C	168
YTH N6-methyladenosine RNA binding protein C1	*YTHDC1*	F: 5′tgcaaggagtgttatcttaa 3′ R: 5′cctgctggaagtacccaatg 3′	XM_027971035.2	60 °C	120
YTH N6-methyladenosine RNA binding protein C2	*YTHDC2*	F: 5′acggtttcatcccacttcagt 3′ R: 5′tccacaaaataccaacacagtga 3′	XM_015096775.3	60 °C	193
YTH N6-methyladenosine RNA binding protein F2	*YTHDF2*	F: 5′caaaaggatggattaaacgatga 3′ R: 5′aagcagcttcacccaaagaa 3′	XM_027965412.2	60 °C	151
YTH N6-methyladenosine RNA binding protein F3	*YTHDF3*	F: 5′ccgccacctcctataaaaca 3′ R: 5′gtctgaggaggcagaactgg 3′	XM_042254248.1	60 °C	101
Tyrosine 3- monooxygenase activation protein zeta	*YWHAZ*	F: 5′ tgtaggatcccgtaggtcatc 3′ R: 5′ ttctctctgtattctcgagcca 3′	NM_001135699	60 °C	168

Relative transcript quantification was performed by real-time polymerase chain reaction (RT-PCR) in a Rotor-Gene Q 5 plex HRM (Qiagen). The PCR was performed in a 15 μL reaction volume containing 7.5 μL 2 × Qiagen PCR Master Mix (Qiagen), 200 nM of each primer (Table 2), and cDNA equivalent to ~ 10 ng RNA. The PCR protocol consisted of two incubation steps (50 °C for 5 min and 95 °C for 2 min), followed by 40 cycles of amplification program (95 °C for 15 s, gene-specific annealing temperature for 30 s, Table 2), a melting curve program (65–95 °C, starting fluorescence acquisition at 65 °C, and taking measurements at 10 s intervals until the temperature reached 95 °C), and finally a cooling step to 4 °C. Fluorescence data were acquired during the elongation step. To minimize handling variation, all samples were analyzed within the same run using a PCR master mix containing all reaction components apart from the sample. The PCR products were analyzed by generating a melting curve to check the specificity and identity of the amplification product. For each primer pair, the efficiency of the PCR reaction was determined by building a standard curve with serial dilutions of a known amount of template, covering at least three orders of magnitude, so that the calibration curve’s linear interval included the interval above and below the abundance of the targets. Only primers achieving an efficiency of reaction between 90 and 110% (3.6 > slope > 3.1) and a coefficient of determination *r*^2^ > 0.99 were used for the analysis.

Target gene normalization for CC samples was performed against the geometrical mean of four housekeeping gene expressions: ribosomal protein L19 (*RPL19*), actin B (*ACTB*), succinate dehydrogenase complex flavoprotein, subunit A (*SDHA*), and tyrosine 3-monooxygenase/tryptophan 5-monooxygenase activation protein zeta (*YWHAZ*). Normalization of oocyte gene expression was performed against luciferase mRNA levels and the number of oocytes per pool [41–43].

### Quantification of m6A RNA methylation in cumulus cells and tissues

#### Tissue collection and total RNA isolation

Ovine tissue including corpus luteum, kidney, liver, lung, ovary, testis, and uterus were collected at a local slaughterhouse. All samples were immediately plunged into RNAlater™ Stabilization Solution (ThermoFisher Scientific) and stored overnight at − 4 °C then at − 20 °C until use.

Total RNA was isolated using 1 mL TRIzol™ Reagent (Invitrogen Corporation, Carlsbad, CA, USA) per 100 mg of tissue and treated with DNase I (Invitrogen Corporation, Carlsbad, CA, USA) according to manufacturer’s protocols. The resulting RNA quantity and purity were checked spectroscopically with NanoDropLite (Fisher Scientific S.A.S., Illkirch Cedex, France).

#### m6A quantification by colorimetric analysis

The methylation status of m6A RNA was detected directly using total RNA isolated from cumulus cells (approximately CCs from 20 COCs per biological replicate) of adult and prepuberal donors at GV and MII stages (*n* = 3 per experimental group) and total RNA isolated from tissues. RNA from cumulus cells was isolated as described above in paragraph “RNA isolation and reverse transcription.”

To detect m6A RNA methylation status directly using total RNA, a colorimetric assay was performed using EpiQuik™ m6A RNA Methylation Quantification Kit (Colorimetric; Epigentek, Farmingdale, NY, USA). The kit is suitable for detecting m6A RNA methylation status directly using total RNA isolated from any species such as mammals, plants, fungi, bacteria, and viruses.

Methylation quantification was analyzed in a 96-well plate using 100 ng of CCs total RNA and 200 ng of tissue total RNA per well. Negative controls and a standard curve covering five orders (range 0.02 to 1 ng of m6A) were analyzed in the same plate. The detected signal was enhanced colorimetrically and then quantified by reading the absorbance at 450 nm with the spectrophotometer SPECTROstar Nano Microplate reader (BMG Labtech, Ortenberg, Germany). Data were analyzed with the Mars® Data Analysis software according to manufacturer’s instructions.

Samples optical density (OD) was used to calculate the percentage of m6A in each sample according to the manufacturer’s instructions. The amount of m6A was calculated with the following equation:

$$\text{m}6\text{A }\left(\text{ng}\right)=\frac{\text{Sample OD}-\text{Negative control OD}}{\text{Slope of the standard curve}}$$

$$\text{m}6\text{A \%}= \frac{\text{m}6\text{A amount }\left(\text{ng}\right)}{S } \times 100\%$$

where *S* is the amount of input sample RNA in ng, and the slope of the standard curve was calculated for each run on the basis of the OD of the standards.

### Statistical analysis

Data were analyzed with GraphPad Prism version 8.0.0 for Windows (GraphPad Software, San Diego, CA, USA). After testing for normality using the Kolmogorov–Smirnov test, gene expression and m6A colorimetric data were analyzed with the general linear model analysis of variance (ANOVA), followed by Tukey’s post hoc comparison when significant differences among groups as a whole were observed. Differences were considered significant when *P* < 0.05.

## Results

### Quantification of m6A RNA methylation in cumulus cells

**Fig. 2:**
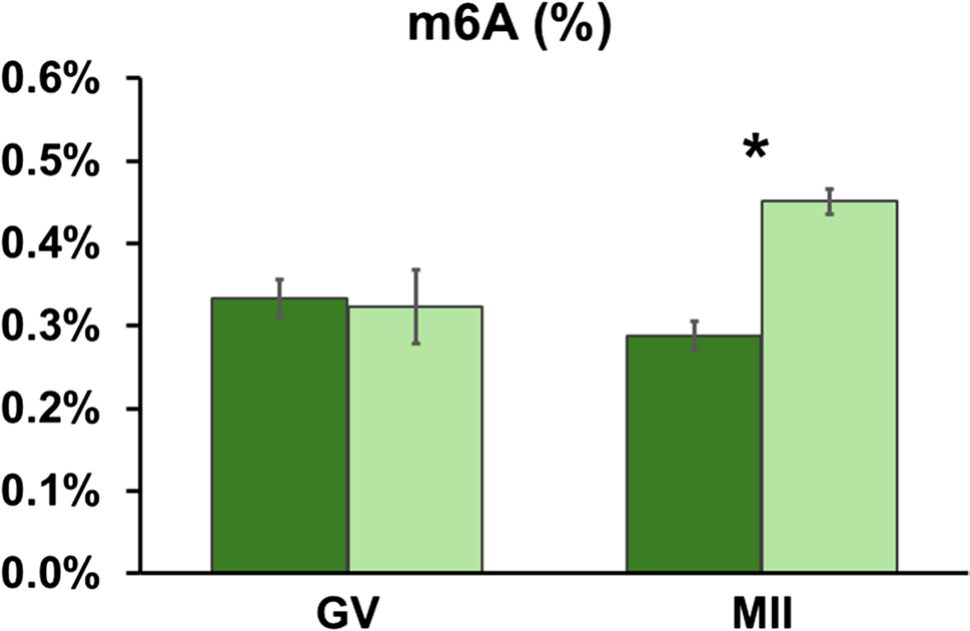
Levels of m6A abundance in cumulus cells of ovine immature (GV) and mature (MII) COCs derived from adult (green column) or prepubertal (light green column) donors. m6A abundance values are expressed as percentage of methylated adenosine in total RNA (%) and show the mean value ± s.e.m. of three replicates for each experimental group (each replicate = 100 ng of total RNA; ANOVA general linear model, * = *P* < 0.05)

Colorimetric assay showed the percentage of methylated adenosine in total RNA of cumulus cells from the GV stage COCs: 0.32% ± 0.4 s.e.m. and 0.33% ± 0.02 s.e.m. in prepubertal and adult donors, respectively (*P* > 0.05). Conversely, the methylated adenosine in total RNA after IVM was higher in CCs of COCs of prepubertal donors (0.45% ± 0.01 s.e.m.) compared to adult sheep (0.29% ± 0.01 s.e.m; *P* < 0.05, Fig. 2).

### Quantification of m6A RNA methylation in reproductive and somatic tissues

**Fig. 3:**
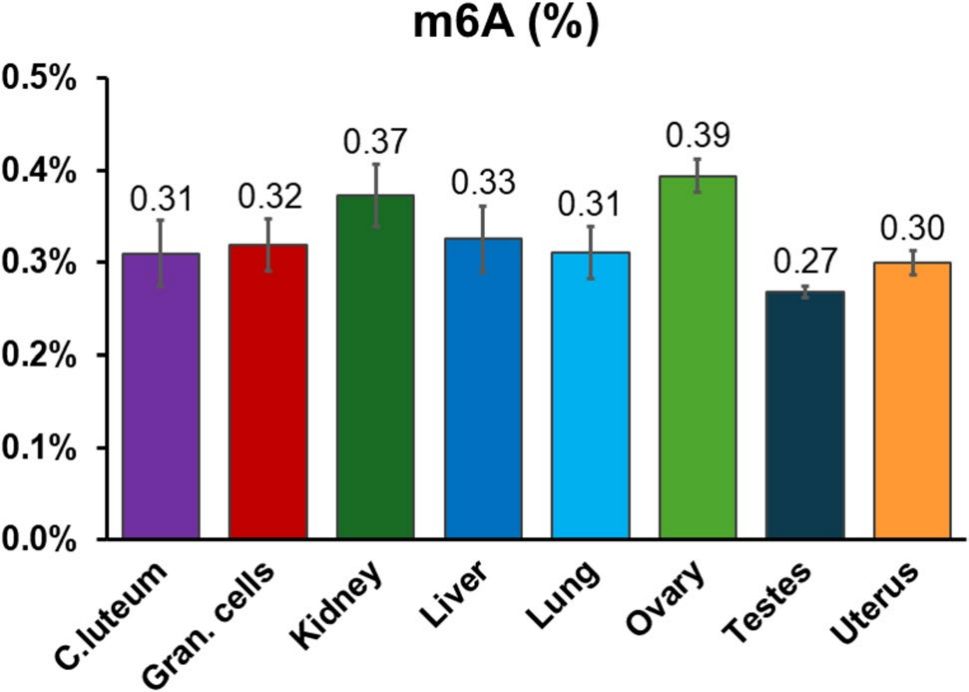
Levels of m6A abundance in adult sheep corpus luteum, granulosa cells, kidney, liver, lung, ovary, testes, and uterus. m6A abundance values are expressed as percentage of methylated adenosine in total RNA and show the mean value ± s.e.m. of three replicates for each tissue (each replicate = 200 ng of total RNA)

To estimate the abundance of m6A in various reproductive and somatic tissues in adult sheep, we analyzed m6A levels in terms of percentage of methylated adenosine in total RNA of corpus luteum, cumulus cells, kidney, liver, lung, ovary, testes, and uterus using a colorimetric assay. The m6A content in these tissues ranged from 0.26 to 0.39% of total RNA (Fig. 3).

### Gene expression analysis

#### Expression of genes encoding m6A methylation-related proteins in oocytes

**Fig. 4:**
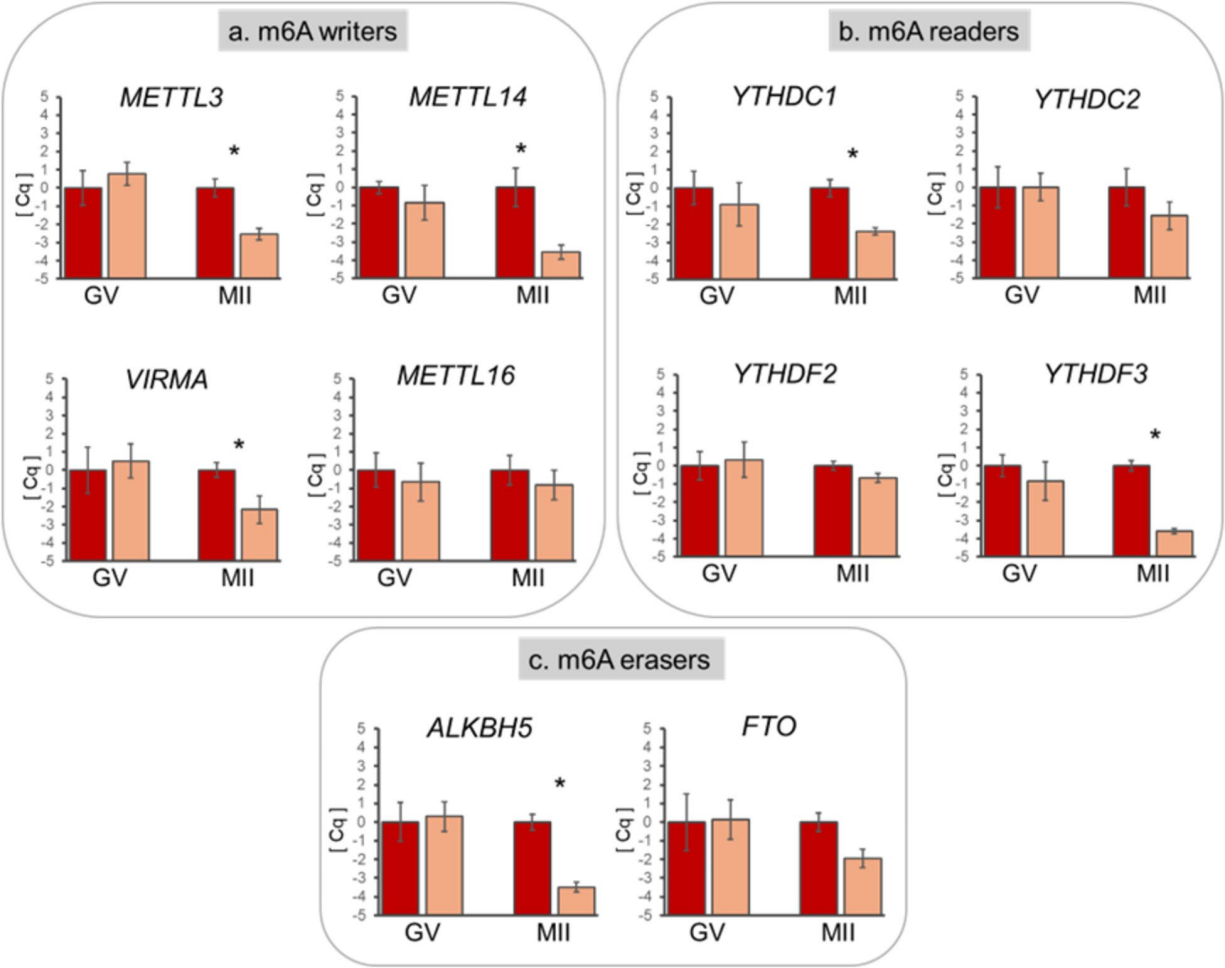
Relative transcript abundance of m6A methylation-related proteins: the writers *METTL3*,* METTL14*,* METTL16*, and *VIRMA* (**a**), the readers *YTHDC1*,* YTHDC2*,* YTHDF2*, and *YTHDF3* (**b**), and the erasers *ALKBH5* and *FTO* (**c**) in ovine immature (GV) and mature oocytes (MII), derived from adults (red column) or prepubertal (yellow column) donors. Relative abundance values are expressed as ΔCq and show the mean value ± s.e.m. of five replicates for each stage (each replicate = pool of ten oocytes). Target gene transcript abundance was normalized against the expression of the exogenous gene Luciferase (Promega). Significant differences were assessed by ANOVA general linear model test; *P < 0.05

In GV oocytes, relative abundance of all transcripts was similar between adult and prepubertal donors. Conversely, after IVM, prepubertal oocytes showed a lower abundance of *METTL3*, *METTL14*, and *VIRMA* (*P* < 0.05; Fig. 4a) and *YTHDC1*,* YTHDF3*, and *ALKBH5* (*P* < 0.05; Fig. 4b and c); while transcript abundance of *METTL16*,* YTHDC2*,* YTHDF2*, and *FTO* showed no differences (Fig. 4a, b, and c).

#### Expression of genes encoding m6A methylation-related proteins in cumulus cells

**Fig. 5:**
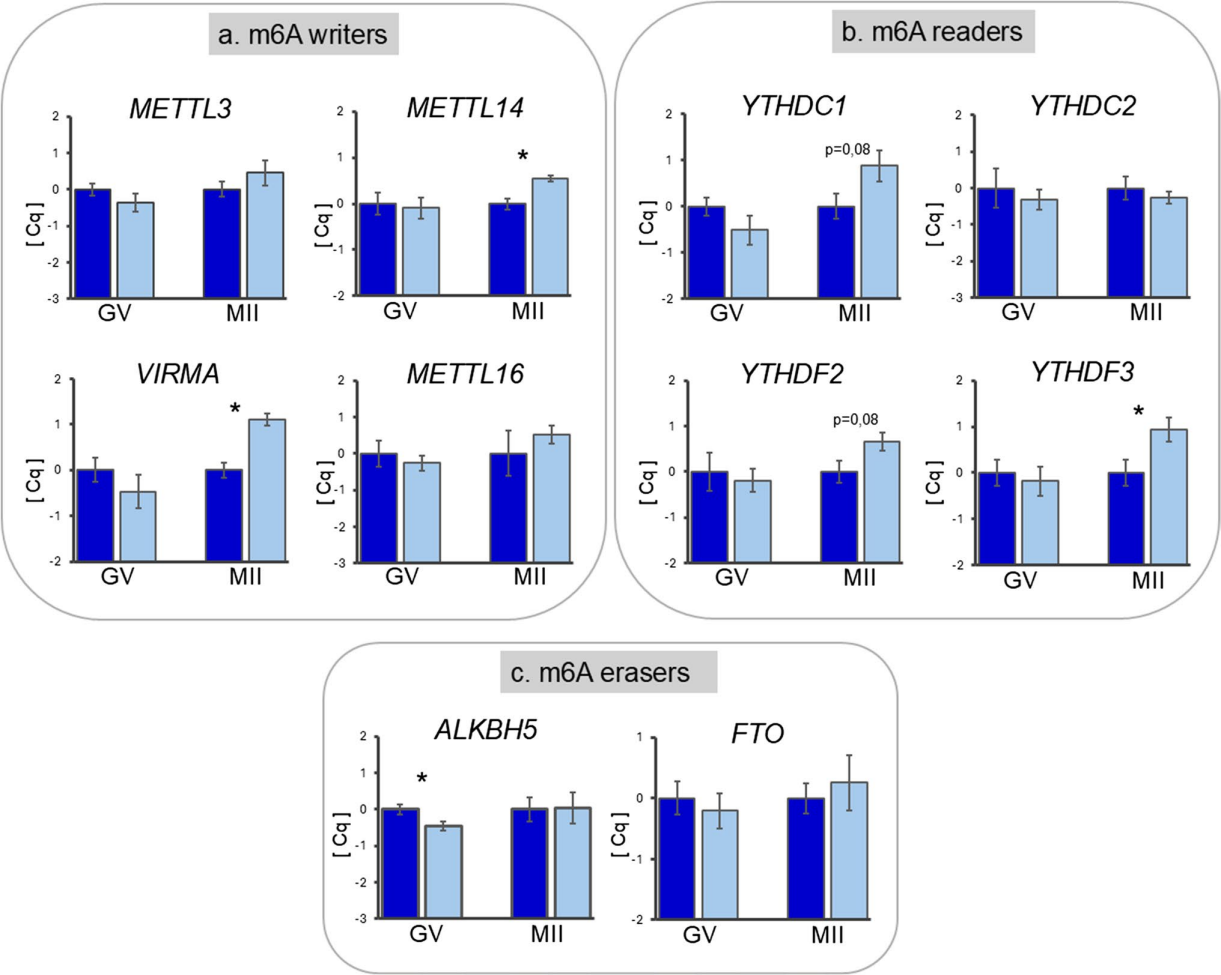
Relative transcript abundance of m6A writers *METTL3*, *METTL14*,* METTL16*, *VIRMA* (**a**); readers *YTHDC1*,* YTHDC2*, *YTHDF2*, and *YTHDF3* (**b**); and erasers *ALKBH5* and *FTO* (**c**) in cumulus cells of ovine immature (GV) and mature COCs (MII) derived from adult (blue column) or prepubertal (light blue column) donors. Relative abundance values are expressed as ΔCq and show the mean value ± s.e.m. of five replicates for each stage (each replicate = CCs from pool of 20 COCs). Target gene transcript abundance was normalized against the expression of the four housekeeping genes *RPL19*, *ACTB*, *YWHAZ*, and *SDHA*. Significant differences were assessed by ANOVA general linear model test; *P ≤ 0.05

Relative transcript abundance was similar in CCs of adult and prepubertal COCs at the GV stage, except for the eraser enzyme *ALKBH5*, which showed a lower abundance in CCs of prepubertal donors (*P* < 0.05; Fig. 5c). Conversely, after IVM, CCs of prepubertal donors showed upregulation of the methyltransferases *METTL14* and *VIRMA* (*P* < 0.05; Fig. 5a) and of the reader *YTHDF3* (*P* ≤ 0.05; Fig. 5b). The readers *YTHDC1* and *YTHDF2* showed an increasing trend in CCs of prepubertal donors (*P* = 0.08; Fig. 5b). Gene expression of *METTL3*, *METTL16*, *YTHDC2*, and *FTO* showed no differences between experimental groups (Fig. 5a, b, and c).

## Discussion

The major finding of the present work is that low competence sheep COCs show alterations in m6A RNA methylation after IVM*.* Alterations include m6A RNA methylation abundance in cumulus cells and transcript dynamics of the m6A methylation-related proteins in both cumulus cells and oocytes. Because of m6A’s crucial role in mRNA post-transcriptional regulation, including maternal transcript degradation, these results support m6A involvement in oocyte developmental potential.

Gene expression regulation in oocytes and embryos is unique due to the transcriptional silence that occurs during oocyte maturation and the early phases of embryo preimplantation development [1, 2]. As a consequence, these developmental stages rely on maternal mRNAs synthesized during oocyte growth and on post-transcriptional mechanisms that ensure the timely availability of specific molecules [1, 2]. The present work studied the patterns of transcript abundance of the key proteins remodeling and reading m6A patterns in sheep oocytes and cumulus cells, which have not been reported before. The expression of writers (*METTL3*, *METTL14*,* METTL16*, and *VIRMA*), erasers (*ALKBH5* and FTO), and readers (*YTHDC1*, *YTHDC2*, *YTHDF2*, and *YTHDF3*) was confirmed in both oocytes and cumulus cells (Figs. 4 and 5), and different gene expression patterns were reported in COCs with high or poor developmental potential, supporting our previous hypothesis that low competence sheep oocytes lack proper gene regulation [38].

Importantly, the transcript abundance of candidate proteins associated with the epitranscriptome exhibited differences in COCs from adult and prepubertal donors after IVM. In both oocytes and cumulus cells, the expression levels of the genes encoding m6A methylation-related proteins are similar in adult and prepubertal donors at the GV stage, in accordance with our previous studies that considered the expression of maternal effect genes, genes involved in DNA methylation, genomic imprinting, pluripotency, and cell cycle regulation within the same model of developmental competence [38, 44]. These data indicate proper transcription and mRNA storage during oocyte growth. We then observed alterations in transcript contents in COCs derived from prepubertal animals after IVM. These findings suggest a differential fate of maternal transcripts (translation or degradation) during IVM in COCs from prepubertal donors, compared to those derived from adult sheep.

In the present study, we observed a lower expression of both m6A writers (*METTL3*, *METTL14*, and *VIRMA*) and readers (*YTHDC1* and YTHDF3) in oocytes of prepubertal donors after IVM (Fig. 4). METTL3 and METTL14 belong to a conserved family of methyltransferases and form a heterodimer complex in the nucleus that catalyzes the methyl transfer to adenosine [45]. METTL3 deficiency in murine oocyte leads to abnormal translation efficiency, probably due to interference in mRNA degradation of genes involved in oocyte maturation and maternal to zygote transition [46]. Moreover, *Mettl3* depletion in murine GV oocyte causes defective polar body extrusion and increased aneuploidy rates during oocyte maturation [21]. The lack of VIRMA, which guides region-selective methylation of the methyltransferase complex [25], causes disorders in spatio-temporal regulation of mouse oocyte gene expression, leading to altered follicular growth and oocyte meiotic maturation [16]. The low transcript abundance of *METTL3*, *METTL14*, and *VIRMA,* we observed in MII oocytes of prepubertal donors, may affect the correct m6A methylation of maternal transcripts and consequently interfere with proper mRNA degradation, leading to altered mRNA dosage. Interestingly, the analysis of expression in CCs reveals an inverse pattern, with a significant increase in *METT14* and *VIRMA* in CC of COCs of prepubertal donors. This indicates a clear difference in gene regulation related to m6A modulation in CC of high- and low-quality COCs.

m6A readers determine the fate of target mRNAs by identifying and interpreting their m6A patterns. Reader proteins are divided into direct and indirect readers according to their ability to directly and specifically combine with m6A. Among the direct readers, a key role is played by a family of highly conserved YTH-domain containing proteins, with several of them playing key roles in mRNA regulation [47]. YTHDC1 is implicated in mRNA exports [27], while YTHDF3 and YTHDF1 facilitate the translation of their target mRNAs before these mRNAs undergo degradation through the YTHDF2-mediated pathway [29]. YTHDF2 interacts with specific polyadenylation factors to initiate transcript deadenylation and induce degradation of m6A-containing mRNAs [7]. Mechanistically, the binding of YTHDF2 to m6A on mRNAs elicits rapid mRNA degradation by recruiting several RNA-degrading enzymes [48]. As a consequence, loss of *YTHDF2* results in the failure to regulate transcript dosage of a cohort of genes during oocyte maturation, leading to embryo arrest in mice and goat [3, 49]. In the present work, we observed a lower *YTHDF3* transcript abundance in prepubertal MII oocytes, which may affect mechanisms of transcript translation or decay.

To provide a comprehensive overview of m6A methylation enzyme expression, we analyzed the mRNA abundance of two erasers: FTO and ALKBH5. They are two members of the AlkB family of non heme Fe (II)/α-ketoglutarate (α-KG) -dependent dioxygenases implicated in demethylating m6a RNA in mammals [30, 50]. The eraser *FTO* did not show any variation in mRNA abundance during IVM in oocytes or CCs, independently of the donor age. Conversely, *ALKBH5* transcript showed a significant increase over maturation in oocytes of adult donors, which was not observed in gametes of young ewes (Fig. 4c). In CCs, a lower transcript abundance of *ALKBH5* was detected before IVM in COCs derived from young donors (Fig. 5c). ALKBH5 controls meiosis-coupled mRNA clearance in oocytes by removing the m6A in mice [10]. Its depletion causes a wide range of defects in oocyte meiosis, including a striking RNA accumulation during meiotic maturation and results in impaired RNA clearance and female infertility [10]. The altered expression we observed in both oocytes and CCs of low competence COCs may significantly impact the m6A methylation of their transcriptomes.

To determine whether the observed changes in mRNA abundance of m6A modulators corresponded to altered levels of m6A in transcripts, m6A in CCs of the differential model was quantified directly using a colorimetric assay. Consistent with the expression data, similar levels of m6A were observed in CCs of oocytes at the GV stage (0.33% and 0.32% of total RNA in CC derived from adult and prepubertal donors, respectively; Fig. 2). However, after IVM, m6A varied depending on COCs competence: CCs of prepubertal oocytes showed higher m6A levels compared to gametes derived from adult donors (0.45% vs 0.29%, *P* < 0.05; Fig. 2). The higher m6A levels in prepubertal-derived CCs may result from the reduced transcript abundance of the m6A eraser *ALKBH5* and from the abnormal persistence of the m6A writers, *METTL14* and *VIRMA*, respectively before and after IVM (Fig. 5). As m6A maintains maternal RNA stability in oocytes, protecting transcripts from degradation [10], abnormally high m6A levels might induce aberrant transcript persistence in cumulus cells. A relation between m6A abundance in CC and developmental competence was previously observed in humans, where higher levels of m6A were found in primary human ovarian granulosa cells (GC) associated with premature ovarian insufficiency [51].

Performing a direct measurement of m6A RNA methylation in oocytes would have been of considerable interest; however, in the present work, we were unable to complete such measurement due to the high number of required oocytes (at least 300 GV per biological replicate, a higher number for MII stage [36]) to obtain the minimum RNA amount (100 ng) necessary for the assay.

As tissue-specific m6A RNA methylation patterns in sheep are largely unknown, we characterized m6A levels in both reproductive and somatic tissues to provide baseline information on the species and for comparison with the m6A abundance observed in cumulus cells (Figs. 2 and 3). The quantification in adult sheep corpus luteum, cumulus cells, kidney, liver, lung, ovary, testes, and uterus showed m6A percentages ranging from 0.27 to 0.39 (Fig. 3). These percentages differ from those observed in pig liver, ovary, and cumulus cells [18]. In human GCs, the level of m6A was lower compared to both sheep and swine (around 0.08% [52]). Such species-specific patterns confirm the existence of unrecognized species-specific layers of transcript regulation, as postulated by Dubuc and co-authors [18]. Nevertheless, post-transcriptional gene regulation by m6A abundance may still benefit from further studies to unveil its general and cell-specific mechanisms.

In summary, this work describes for the first time the dynamics of m6A in sheep COCs and highlights alterations in oocytes and cumulus cells derived from prepubertal donors. More importantly, we show that dysregulations arise after IVM, supporting the hypothesis that the molecular endowment of oocytes with low developmental competence hinders proper gene regulation during the transcriptional silence that encompasses oocyte maturation and early embryo development.
